# A 25-Year-Old Chronic Ketamine User with Urinary Symptoms; a Case Report

**DOI:** 10.22037/aaem.v10i1.1449

**Published:** 2022-01-31

**Authors:** Chin-Chu Wu, Aming Chor-Ming Lin

**Affiliations:** 1 Department of Medical Imaging, Shin Kong Wu Ho-Su Memorial Hospital, Taipei, Taiwan.; 2 Emergency Department, Shin Kong Wu Ho-Su Memorial Hospital, Taipei, Taiwan.; 3 School of Medicine, Fu-Jen Catholic University, New Taipei City, Taiwan.

**Keywords:** Tomography, X-Ray Computed, Ketamine, Urologic Diseases, Urinary Tract Infections

## Abstract

Ketamine is mainly used for short-acting general anesthesia, chronic pain, sedation, depression, and bipolar disorder. Long-term ketamine use may cause lower urinary tract symptoms and voiding dysfunction. Small capacity and fibrotic bladder can be associated with chronic ketamine use. Here, we present a 25-year-old male with a history of chronic ketamine use complicated with contracted heart-shape bladder.

## 1. Introduction:

Sustained ketamine use results in small capacity, and fibrotic and contracted bladder, which can lead to damage to the urinary tract system. The clinical presentations range from suprapubic pain, small volume voids, and irritative lower tract symptoms to painful hematuria. Long-term abusers with a small and fibrotic bladder and poor compliance are at high risk for complications including hydronephrosis, vesicoureteral reflux, urolithiasis, chronic renal failure, and recurrent urinary tract infections (1). We herein report a case of chronic ketamine use, complicated with contracted bladder with a heart-shape appearance on computed tomography (CT) scan.

## 2. Case presentation:

**Figure 1:**
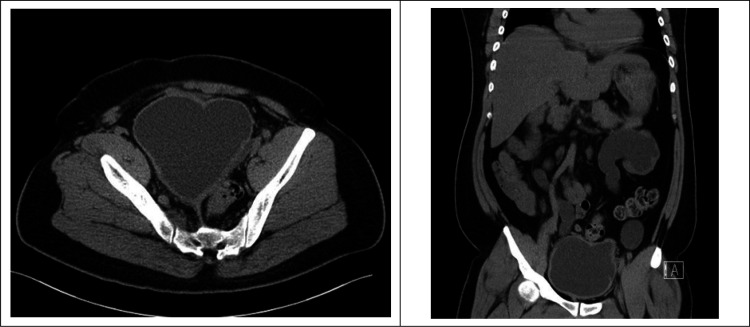
Axial (left) and coronal (right) views of the patient’s abdominopelvic computed tomography scan

A 25-year-old man presented to the emergency department with complaint of fever, urinary frequency, dysuria, bilateral dull flank pain, and unspecific abdominal pain for one week. He had a history of bipolar disorder and chronic ketamine use. On arrival, his blood pressure was 138/82 mmHg, with a heart rate of 101 beats/minute, a respiratory rate of 20 beats/minute, and oxygen saturation of 98% on room air. His temperature was 38.2 °C. On physical examination, the patient had lower abdominal tenderness and bilateral flank knocking pain. The rest of physical examination were unremarkable. The complete blood cell count showed the following results: leukocyte count 14500/mm3 with 92% of segmented neutrophils, hemoglobin 10.8 gram/deciliter, platelet 520000/microliter, and an international normalized ratio (INR) of 0.8. Urinalysis showed severe pyuria and gross hematuria. Other laboratory findings included: glucose 100 milligram/deciliter, blood urea nitrogen (BUN) 38 mg/dl, serum creatinine 2.6 mg/dl, sodium 135 mEq/L, potassium 4.1 mEq/L, and serum glutamic oxaloacetic transaminase (SGOT) 48 U/L. The patient underwent abdominopelvic computed tomography (CT) scan without contrast material due to renal insufficiency. Axial view of abdominopelvic CT scan showed thick-wall bladder with pseudo-diverticula, dilatation of bilateral proximal ureter, and left hydronephrosis (figure 1). Coronal view of CT scan showed a contracted heart-shape bladder (figure 1). A diagnosis of ketamine-associated uropathy, complicated with urinary tract infection, was made. The patient was started on intravenous fluids, parenteral antibiotics, and continuous urinary drainage through a Foley. On the following days, urine and blood cultures yielded Escherichia coli. He recovered with conservative management and was discharged 12 days later. 

## 3. Discussion:

Ketamine is a non-competitive N-methyl-D-aspartate receptor antagonist medication, mainly used for short-acting general anesthesia, chronic pain, sedation, depression, and bipolar disorder (2). Ketamine is also used as a recreational drug due to its hallucinogenic and dissociative effects. Because of its low price and easy usage, use of ketamine as a recreational drug is being increasingly reported worldwide. Long-term ketamine abuse may cause severe lower urinary tract symptoms and voiding dysfunction. Chronic ketamine use can damage many organs including the brain, heart, liver, gastrointestinal tract, and genitourinary system (3). Urinary tract abnormalities are the most commonly reported undesirable chronic effects related to ketamine abuse (4, 5). Chronic ketamine abuse may be associated with ulcerative cystitis, urge incontinence, decreased bladder volume, decreased bladder compliance, detrusor overactivity, and painful hematuria (6). Secondary renal damage can occur in long-term abusers (7, 8). Lower urinary tract symptoms may be associated with chronic urinary infection and ketamine-associated ulcerative cystitis. Cessation of ketamine use and proper treatment may improve most lower urinary tract symptoms. 

## 4. Conclusion:

Patients with a history of chronic ketamine use with persistent lower tract symptoms need to be encouraged to immediately cease its use and refer to a urologist for assessment. Contracted bladder patients with frequent recurrent urinary tract infection should be investigated. CT is useful in detecting the causes of urinary symptoms, such as calculi, bladder debris, bladder fibrosis, and poor bladder compliance, as well as in early diagnosis of ketamine-associated uropathy. 

## 5. Declarations:

### 5.1 Acknowledgements

We acknowledge all the staff of department of diagnostic radiology and emergency department of Shin Kong Wu Ho-Su Memorial Hospital, Taipei, Taiwan. 

### 5.2 Conflict of interest

None

### 5.3 Funding and support

None

### 5.4 Authors’ contributions

All authors passed four criteria for authorship contribution based on recommendations of the Internal Committee of Medical Journal Editors.
